# Functional Characterization of a Novel Homozygous *DNAH5* Single-Nucleotide Intronic Deletion in a Consanguineous Portuguese Family with Primary Ciliary Dyskinesia

**DOI:** 10.3390/cells15111022

**Published:** 2026-06-02

**Authors:** Catarina Hilário, Sara Raimundo, Catarina Dias, Joana Saramago, Telma Oliveira, Rute Pereira, Sofia Quental, João Parente Freixo, Luís Gales, Jorge Oliveira, Rosália Sá, Mário Sousa

**Affiliations:** 1 Department of Pulmonology, Unidade Local de Saúde de Trás-os-Montes e Alto Douro, Hospital de Vila Real, Avenida da Noruega, Lordelo, 5000-508 Vila Real, Portugal; acshilario@chtmad.min-saude.pt; 2 Department of Pulmonology, Unidade Local de Saúde de Santo António, Largo Professor Abel Salazar, 4099-001 Porto, Portugal; catarinadias1@gmail.com; 3 UMIB-Unit for Multidisciplinary Research in Biomedicine, University of Porto, Rua de Jorge de Viterbo Ferreira, 4050-346 Porto, Portugal; jfsaramago@icbas.up.pt (J.S.); tsoliveira@icbas.up.pt (T.O.); rrpereira@icbas.up.pt (R.P.); jmoliveira@ibmc.up.pt (J.O.); rmsa@icbas.up.pt (R.S.); 4 ITR-Laboratory for Integrative and Translational Research in Population Health, University of Porto, Rua das Taipas 135, 4050-600 Porto, Portugal; 5 Laboratory of Cell Biology, Department of Microscopy, ICBAS-School of Medicine and Biomedical Sciences, University of Porto, Rua de Jorge de Viterbo Ferreira, 4050-346 Porto, Portugal; 6 ANI-Agência Nacional de Inovação, Rua de Salazares 842, 4149-002 Porto, Portugal; 7 IPATIMUP-Institute of Molecular Pathology and Immunology, University of Porto, Rua Júlio Amaral de Carvalho 45, 4200-135 Porto, Portugal; mquental@ipatimup.pt; 8 i3S-Institute for Investigation and Innovation in Health, University of Porto, Rua Alfredo Allen, 4200-135 Porto, Portugal; joao.freixo@ibmc.up (J.P.F.); lgales@ibmc.up.pt (L.G.); 9 CGPP-Center for Predictive and Preventive Genetics, IBMC-Institute of Molecular and Cell Biology, University of Porto, Rua Alfredo Allen, 4200-135 Porto, Portugal; 10 Department of Chemistry, ICBAS-School of Medicine and Biomedical Sciences, University of Porto, Rua de Jorge de Viterbo Ferreira, 4050-346 Porto, Portugal; 11 Bioengineering and Synthetic Microbiology Group, IBMC-Institute of Cellular and Molecular Biology, University of Porto, Rua Alfredo Allen, 4200-135 Porto, Portugal; 12 Centro Hospitalar Universitário de São João, Unidade Local de Saúde de São João, Alameda Professor Hernâni Monteiro, 4200-319 Porto, Portugal

**Keywords:** primary ciliary dyskinesia, bronchiectasis, DNAH5, variant of uncertain significance, high-speed videomicroscopy, transmission electron microscopy, genetic screening, immunofluorescence, mRNA sequencing

## Abstract

**Highlights:**

**What are the main findings?**

- A male with respiratory dysfunction of the primary ciliary dyskinesia type presented a novel homozygotic *DNAH5* variant, total cilia immotility, predominant absence of outer dynein arms, and concomitant markedly absent protein level. Family confirmed homozygosity and co-segregation with the phenotype.

**What is the implication of the main finding?**

- Results enabled us to reclassify the variant as pathogenic, enabling its clinical actionability. As the patient also presented with oligoteratozoospermia, with markedly absent protein level but unaffected motility, results further support that this variant impacts differently in the respiratory and reproductive cells.

**Abstract:**

Primary ciliary dyskinesia (PCD) is a rare genetic disorder mainly characterized by impaired mucociliary clearance and chronic respiratory symptoms. From a consanguineous family, a male patient, although with respiratory complaints since birth, was diagnosed with PCD only in adulthood. Whole-exome sequencing disclosed a novel homozygous intronic single-nucleotide deletion, NM_001369.3(*DNAH5*):c.13723+4del, initially classified as of uncertain clinical significance. Digital highspeed videomicroscopy (HSVM) evidenced a null ciliary beating frequency; transmission electron microscopy showed absence of outer dynein arms (class-1); and immunofluorescence (IF) demonstrated markedly absent DNAH5 protein level in the apical cilia region with delocalization to the transition and basal-body regions. Bioinformatic analysis predicted altered splicing at the donor splice site of exon 78, whereas mRNA sequencing revealed two splicing defects: the mainly expressed transcript corresponding to exon 78 skipping and a minor transcript originated from a cryptic splice site in exon 78. The patient was infertile and showed severe oligoteratozoospermia. Sperm IF analysis revealed absence of DNAH5 from the flagellum with accumulation at the neck region. The family study confirmed homozygosity. The present results support a pathogenic role for the c.13723+4del variant and underscore the importance of integrating clinical, ultrastructural, DNA, mRNA and protein analyses to clarify and contribute to PCD diagnosis.

## 1. Introduction

Primary ciliary dyskinesia (PCD, ORPHA:244) is a rare, genetically heterogeneous disorder with an estimated prevalence that varies across ethnic groups, i.e., 1:15,000–1:30,000 live births [1], 1:15,000 [2], 1:10,000 [3], or 1:7500 [4], and often remaining underdiagnosed. PCD is predominantly an autosomal recessive disorder [5], being rarely autosomal dominant [6,7] or X-linked [8,9].

PCD is characterized by dysfunction of motile cilia. The motor apparatus of cilia (axoneme) is structurally complex, rendering possible multiple genetic variants. This genetic variability justifies the heterogeneous phenotype and absence of a single diagnostic test. Due to the heterogeneous clinical presentation, the disease is prone to misdiagnosis or underdiagnosis [10], making diagnosis difficult and requiring several differential screenings. In consequence, PCD diagnosis is often delayed towards adult life, postponing target treatments that should be implemented sooner [11].

Motile cilia beat rhythmically [12] and are present is several tissues [13]. In the fetal node, they are responsible for establishing normal laterality [14], with approximately half of PCD patients exhibiting laterality defects, and a subset presenting the classical triad of *situs inversus*, chronic sinusitis, and bronchiectasis, defining the Kartagener syndrome [3,15,16]. In the respiratory tract, cilia are found in the Eustachian tube, sinuses, nose, and bronchi, affecting the mucociliary clearance (MCC) and thus prompting to chronic inflammatory and infections. PCD usually manifests from birth, often with unexplained neonatal respiratory distress, or in early childhood, with persistent wet cough, chronic nasal and sinus congestion, recurrent otitis media, and bronchiectasis [5]. Male subfertility has been reported in about 81% of the PCD cases [17], since the spermatozoon flagellum shares with cilia the same motility apparatus [18]. Female subfertility is also associated with PCD in about 61% of the cases [17,19]. Here, dysfunctional cilia were suggested to impair fallopian tube embryo transfer [20,21] and to interfere with implantation due to the interaction of the blastocyst with endometrium cilia [22]. Finally, cilia abnormalities in the cerebrum ventricular ependyma [6] lead to the development of hydrocephalus in 6% of the cases due to deficient fluid flow [7,23].

For managing PCD diagnosis, the guidelines of the European Respiratory Society (ERS) and of the American Thoracic Society (ATS) recommend a stepwise diagnostic approach integrating clinical features and specialized testing [15,24,25]. These include nasal nitric oxide (nNO), which, when persistently low, supports the diagnosis [26,27,28]; high-speed videomicroscopy (HSVM), which gives information on cilia beating frequency and pattern [29]; transmission electron microscopy (TEM), which assesses ultrastructural defects and can be diagnostic [30]; genetic testing, mainly by whole-exome sequencing (WES) or whole-genome sequencing, which has become central to PCD diagnosis and is considered a first-line investigation [31,32]; and immunofluorescence (IF), which enables detection of decreased, absent, or mislocalized ciliary proteins [33,34].

Genetic testing plays a central role in the diagnostic process, with more than 55 disease-related genes currently associated with PCD and increasingly well-described genotype–phenotype correlations. Nevertheless, pathogenic variants remain unidentified in approximately 20–30% of patients. Furthermore, due to the marked genetic heterogeneity of PCD, variants of uncertain significance (VUS) are frequently detected, requiring careful interpretation in the context of clinical presentation and functional studies to determine their pathogenic relevance and enable reclassification [25].

Herein, we report the case of a patient diagnosed with PCD in adulthood, despite longstanding symptoms since childhood, in whom a VUS was identified in a gene associated with PCD. The male patient was born from a consanguineous family and carried, in homozygosity, a novel *DNAH5* frameshift variant, NM_001369.3(*DNAH5*):c.13723+4del, which was classified as of unknown significance. The patient has no *situs inversus*, and semen analysis revealed oligoteratozoospermia, with preserved sperm motility. HSVM results showed an immotility cilia pattern; TEM analysis revealed absence of the outer dynein arms (class 1); and IF evidenced markedly absent protein level, with delocalization, suggesting a defect on dynein assembly. Sequencing of mRNA confirmed altered splicing, with major transcripts revealing exon skipping and minor transcripts revealing the introduction of a splicing cryptic site. The family study confirmed variant homozygosity, with the other family members depicting normal ciliary function. Results support a pathogenic role for the NM_001369.3(*DNAH5*):c.13723+4del variant. This case highlights the diagnostic challenges posed by atypical or delayed presentations and underscores the importance of a comprehensive, multidisciplinary approach integrating clinical assessment with multiple complementary diagnostic modalities.

## 2. Materials and Methods

### 2.1. Ethical Considerations

All procedures were conducted in accordance with applicable ethical standards and institutional protocols. Patient records were reviewed in accordance with confidentiality protocols to obtain comprehensive data, including demographic characteristics, clinical and microbiological data, pulmonary function, imaging studies, and diagnostic exams, according to Hospital rules. All participants provided written informed consent for the collection and use of both clinical data and biological material for research purposes, in accordance with the approval granted by the Joint Ethics Committee of the Hospital and University, CHUP/ICBAS approval number 2020-094 (077-DEFI-078-CE), of 21 May 2020. This work did not involve human or animal experiments, and thus the provisions of the Declaration of Helsinki as revised in Tokyo 2004 do not apply to this work.

### 2.2. Patient

The patient, offspring of a consanguineous couple, presented a PCD phenotype, and the genetic study revealed a novel homozygotic frameshift variant of unknown clinical significance in the *DNAH5* gene, *DNAH5* NM_001369.3(*DNAH5*):c.13723+4del.

### 2.3. Nasal Sample Collection

Nasal brushes were performed by a medical specialist after full evaluation of the patient for the technique. The patient was not submitted to nasal brush during acute disease episode and the day before he stopped any medication, according to the guidelines of the ERS and of the ATS [15,24,25]. Nasal cells were obtained by nasal brushing from the proband and family, using a cytology soft sterile brush (Endobrush, Biogyn SNC, Mirandola, Italy) [35,36]. Cells of one nostril were placed in culture medium for high-speed video microscopy (HSVM). Cells from the other nostril were processed for transmission electron microscopy (TEM). Family nasal ciliary cells were not used for TEM analysis. A second sample collection was performed months later for immunofluorescence (IF) and mRNA studies.

### 2.4. Ciliary Beat Frequency and Beat Patterns by Digital Highspeed Videomicroscopy

Ciliary beat frequency (CBF) and ciliary beat pattern (CBP) analysis was performed as previously described [29]. Collected cells were placed in Medium-199 at 37 °C until analysis. Cell imaging was executed in an inverted microscope (Olympus CKX41, Nikon, Tokyo, Japan), with beating ciliated cells being recorded using a digital high-speed video camera (Semiconductor Vita 5000, Pixelink/Navitar, Inc., New York, NY, USA) at a rate of 300–400 frames per second. CBF was quantified using a semi-automated method, the CiliarMove program [37]. In total, 20 × 3 ciliary regions, from proband and parents, were examined. ImageJ, version 1.54g [38] was used for CBP quantification. Normal beating pattern was defined by a coordinated ciliary beat in a back-and-forth movement along the entire epithelial edge. Abnormal beating patterns were categorized into eight distinct dyskinetic CBPs (normal; coordinated, with low amplitude; coordinated and partially immotile; uncoordinated, with low amplitude; hyperkinetic; hyperkinetic, with low amplitude; circular; and total immotile) [39,40].

### 2.5. Transmission Electron Microscopy

Proband nasal samples were fixed with 2.5% glutaraldehyde (Sigma-Aldrich, St. Louis, MO, USA) in 0.1 M cacodylate buffer (Merck, Darmstadt, Germany), at pH 7.2, for 2 h at room temperature (RT). Cells were then post-fixed with 2% osmium tetroxide (Merck) in the same buffer for 2 h at 4 °C. Following dehydration in a graded ethanol series (VWR, Radnor, PA, USA), cells were treated with 1% tannic acid (Merck) in 100% ethanol, and finally embedded in epoxy resin (Epon, Sigma-Aldrich). Ultramicrotomy was performed on an LKB-ultramicrotome (Leica Microsystems, Wetzlar, Germany), using diamond knives (Diatome, Quackertown, PA, USA). Semithin sections were stained with methylene blue-Azur II (Merck). Ultrathin sections, retrieved on copper grids (Taab, Berks, UK), were double-contrasted with aqueous uranyl acetate (BDH, Poole, UK) and lead citrate (Merck), and observed on a JEOL 100CXII transmission electron microscope (JEOL, Tokyo, Japan), operated at 60 kV [35,36].

### 2.6. Ultrastructural Determination of Morphological Classes

Based on systematic defects in any of the axonemal structures [41], diagnosis of Class 1 and Class 2 ultrastructural defects followed the international consensus guideline for reporting transmission electron microscopy results in the diagnosis of PCD (BEAT PCD TEM Criteria) [30].

In these criteria, the axoneme structure is presented in three distinct morphological classes, class-1 defects (indicative of PCD), class-2 defects (suggestive of PCD), and class-normal (normal axoneme structure), requiring the analysis of at least 50 axonemes in high-magnification cross-sections that show complete ciliary membrane and intact ciliary structure (54 sections for axoneme/dynein arms, 143 sections for basal bodies, and 167 sections for central pair defect analysis, in the present case). Class-1 defects confirm the diagnosis of PCD and include defects in ODA, combined defects in ODA and IDA, and disorganization of axonemal microtubules with absence of IDA. Class-2 defects only indicate a diagnosis of PCD when associated with other complementary tests (HSVM, IF, or genetics), and they include the number of ciliated cells, central pair defects, basal-body mislocalization, disorganization of axonemal microtubules with absence of IDA, absence of ODA in 25–50% of the cross-sections, and combined absence of ODA and IDA in 25–50% of the cross-sections [30].

### 2.7. Ultrastructural Determination of Ciliary Beat Axis

Ciliary beat axis and the ciliary deviation were evaluated as described [42]. For that, in cross-sections with intact axonemes, a blue line was drawn parallel to the central microtubules in each ciliary axoneme. Based on the main orientation of the blue lines, a reference red line was then drawn. This red line is perpendicular to the blue lines and passes between the two microtubules of the central pair of a chosen cilium. The angle of each blue line to the reference line is then calculated. Angles are then subtracted from their mean. The mean of the subtracted angles is equal or lower than zero. The standard deviation (SD) of the subtracted angles corresponds to the ciliary beat axis and ciliary deviation. In these criteria, cilia movement orientation is presented as three distinct types, PCD-type (cilia orientation compatible with the distribution described in patients with ciliary movement disorder such as primary ciliary dyskinesia), BQ-type (cilia orientation compatible with the distribution described in patients with ciliary movement disorder such as bronchiectasis), and Normal-type (cilia orientation compatible with the distribution described in healthy patients).

### 2.8. Quantitative TEM Determination of the Inflammation Degree

In a minimum of 200 cross-sections (766 in the present case), cilia were quantified regarding the presence of inflammation signs, which included blebs (membrane protrusions and enlargement of the cytoplasmic space), compound cilia (several axonemes in the same cilium), naked cilia (absence or poor definition of the cilium membrane, with three degrees of severity: <25%, 25–50%, and >50%), and total axoneme microtubule disarrangement [43,44].

### 2.9. Nucleic Acid Extraction

Peripheral blood from proband, both parents, and sister was collected in EDTA tubes (VACUETTE, Porto, Portugal). Genomic DNA was extracted from peripheral blood leukocytes, using the QIAsymphony system designed for automated purification from human whole blood.

### 2.10. Whole-Exome Sequencing

Whole-exome sequencing (WES) was performed using the ExomeXtra^®^ capture kit (CeGaT, Tübingen, Germany), performed on a NovaSeq 6000 system (Illumina, San Diego, CA, USA). Variant analysis was restricted to a virtual multigene panel of 58 genes associated with primary ciliary dyskinesia and differential diagnosis: *ACVR2B*, *BRWD1*, *CCDC103*, *CCDC39*, *CCDC40*, *CCDC65*, *CCNO*, *CENPF*, *CFAP298*, *CFAP300*, *CFAP74*, *CLXN*, *DAW1*, *DNAAF1*, *DNAAF11*, *DNAAF2*, *DNAAF3*, *DNAAF4*, *DNAAF5*, *DNAAF6*, *DNAH1*, *DNAH11*, *DNAH5*, *DNAH7*, *DNAH8*, *DNAH9*, *DNAI1*, *DNAI2*, *DNAJB13*, *DNAL1*, *DRC1*, *FOXJ1*, *GAS2L2*, *GAS8*, *HYDIN*, *INVS*, *LRRC56*, *MCIDAS*, *NEK10*, *NME5*, *NME8*, *ODAD1*, *ODAD2*, *ODAD3*, *ODAD4*, *OFD1*, *RPGR*, *RSPH1*, *RSPH3*, *RSPH4A*, *RSPH9*, *SPAG1*, *SPEF2*, *STK36*, *TP73*, *TTC12*, *ZIC3*, and *ZMYND10.* Variants were filtered by their minor allele-frequency (MAF below 1% in population databases: NCBI’s dbSNP, 1000 Genome Project, Exome Variant Project, ExAC and gnomAD) for those that resulted in a change at the protein level and/or previously described in NCBI’s ClinVar.

### 2.11. Sanger Sequencing

To perform variant segregation analysis in the family, specific primers were designed to amplify the genomic regions encompassing the variant identified in the proband: *DNAH5*_Ex78_F: 5′-GTAAAACGACGGCCAGTTGTACGTCCTTCGAGTTAGAGTG-3′; *DNAH5*_Ex78_R: 5′-CAGGAAACAGCTATGACCAACATGGGTGTGAATGTAAAGC-3′.

After PCR amplification products were purified with Exo/SAP Go (GRiSP, Porto, Portugal) and sequenced by Sanger sequencing, using Big Dye Terminator Cycle Sequencing v1.1 (Thermo fisher Scientific, Waltham, MA, USA), they were analyzed on an ABI 3500 Dx Genetic Analyzer (Thermo fisher Scientific, USA). Sequencing results were analyzed in SeqScape software version v3.0 (Thermo fisher Scientific, USA).

### 2.12. mRNA Sequencing

Nasal epithelial cells were collected from the patient and three healthy controls by gentle rotation of a nasal swab against the nasal epithelium. Swabs were immediately immersed in RNAlater (Thermo Fisher Scientific), and total RNA was isolated using the Maxwell^®^ RSC Instrument and the Maxwell^®^ RSC simplyRNA Tissue Kit (Promega, Madison, WI, USA), following the manufacturer’s protocol. Minor modifications were introduced during the pretreatment step to remove excess RNAlater, prior to purification. Briefly, samples were centrifuged at 350× *g* for 4 min, and the supernatant was carefully removed to avoid disturbing the cell pellet. Cell pellets were resuspended in 200 µL of Thioglycerol/Homogenization Solution, followed by the addition of 200 µL of Lysis Buffer and 25 µL of Proteinase K. Samples were incubated at 56 °C for 1 h (Maxwell^®^ RSC simplyRNA Tissue Kit, Promega), after which automated RNA extraction proceeded according to the manufacturer’s recommendations. Complementary DNA (cDNA) was synthesized from total RNA using the SuperScript™ VILO™ cDNA Synthesis Kit (Invitrogen, Waltham, MA, USA), following the manufacturer’s instructions. Two PCR assays were performed to amplify *DNAH5* (NM_001369.3) regions spanning exons 76–79 and 77–79. The following primer pairs were used, respectively: F: TTGGTGGAAAAAAGCTTCTTGGATT and R: ACACTCCCCACATGTTACTTGACA (565 bp); and F: TTTACCTCGTGGGTTTTCAATGG and R: TGTTCAGGGGTCTGGGCTGT (431 bp).

PCR products were purified and sequenced bidirectionally using the PCR primers and the BigDye^®^ Terminator Cycle Sequencing Kit (Applied Biosystems, Waltham, MA, USA) on an ABI PRISM^®^ 3730XL DNA Sequencer (Applied Biosystems).

### 2.13. Quantitative Immunofluorescence

Protein spatial localization and protein signal intensity analysis of nasal epithelial cells was performed as previously described [33,34,36,45]. Cells were spread onto glass slides (LABSOLUTE, Renningen, Germany), air-dried, and stored at −80 °C until use. Fixation was performed with 4% paraformaldehyde (20 min, RT) (Merck) in PBS at pH 7.4 (PAN-Biotech, Aidenbach, Germany), and then permeabilized with 0.2% Triton X-100 (Sigma-Aldrich) (15 min, RT) in PBS, followed by antigen blockage with 5% non-fat milk (60 min, RT) (Nestlé, Vevey, Switzerland). Cells were incubated overnight at 4 °C with primary antibody rabbit anti-DNAH5 (dynein axonemal heavy chain 5; HPA037470; Atlas Antibodies, Stockholm, Sweden) (for outer dynein arms), rabbit anti-DNALI1 (dynein axonemal light intermediate chain 1; HPA028305: Atlas Antibodies, Stockholm, Sweden) (for inner dynein arms), rabbit anti-GAS8 (growth arrest specific 8; HPA041311; Atlas Antibodies, Stockholm, Sweden) (for nexin–dynein regulatory complexes), rabbit anti-RSPH9 (radial spoke head component 9; HPA031703; Atlas Antibodies, Stockholm, Sweden) (for radial spokes), and mouse anti-acetylated α-tubulin (6-11B-1; Santa Cruz Biotechnology, Dallas, TX, USA) (for axonemal microtubules). For each experiment, a negative control, through the omission of the primary antibody, was included. Goat anti-Rabbit IgG (H+L) Cross-Adsorbed Secondary Antibody, Alexa Fluor™ 488: A-11008, and CoraLite594-conjugated Goat Anti-Mouse IgG (H+L) (Proteintech, Rosemont, IL, USA) were used as secondary antibodies. Counterstaining was performed with Vectashield antifade mounting medium, containing 4′,6-diamidino-2-phenylindole (DAPI, Vector Laboratories, Newark, CA, USA). Cell imaging was executed in an epifluorescence microscope (Eclipse E400; Nikon, Tokyo, Japan), with the corrected total cell fluorescence (CTCF) being calculated according to the formula CTCF = integrated density − (selected cell area × mean fluorescence of background readings). Statistical analyses for the proband and family members were performed using GraphPad Prism, Version 8.0.2, applying the Mann–Whitney test and the Kruskal–Wallis test, respectively. Statistical significance levels were defined as alpha < 0.05.

### 2.14. Structural Protein Analyses

Structural models obtained by X-ray crystallography and cryo-electron microscopy from multiple sources and available in the Protein Data Bank were analyzed. These included models of human and DNAH5 dynein axonemal heavy chains, as well as non-human axonal structures.

## 3. Results

### 3.1. Patient and Family Characteristics

The proband, although diagnosed at the age of 23 years, presented since childhood daily productive cough and recurrent respiratory infections, leading to multiple hospitalizations between ages of 6 and 15. He was born at 39 weeks of gestation from an eutocic delivery (normal gestation), and presented at birth 3.240 kg, measured 50 cm, with an Apgar of 10/10.

Physical examination of the chest and transthoracic echocardiography revealed no laterality defects nor structural heart disease. Albeit pulmonary auscultation remained normal between respiratory exacerbations, he progressively developed persistent productive cough, chronic otitis media, chronic rhinosinusitis with nasal polyposis and persistent rhinorrhea, mild intermittent non-allergic asthma, exertional dyspnea, and occasional wheezing. He referred professional exposure to flour and oil.

In 2019, the patient experienced an episode of infectious mononucleosis, and in 2023, he was hospitalized due to acute pericarditis. He has a normal body mass index (24 kg/m^2^), is a non-smoker, and reports beekeeping as a leisure activity, with no other relevant environmental or occupational exposures.

Repeated sputum cultures isolated *Streptococcus pneumoniae* and *Haemophilus influenzae* (January 2023), *Haemophilus influenzae* (October 2023 and November 2024), *Haemophilus influenzae* and *Moraxella catarrhalis* (December 2024), and *Moraxella catarrhalis* (October 2025). Each infectious episode was treated with appropriate antibiotic therapy, usually resulting in clinical improvement. The patient is currently managed with nebulized hypertonic saline; bronchodilators, as needed; respiratory physiotherapy; and airway clearance exercises.

The patient was born to consanguineous parents (first cousins). The father, aged 52 years, has no history of respiratory disease. The mother, aged 50 years, in the context of isolated IgA deficiency, experiences chronic rhinitis and recurrent episodes of bronchitis. The sister (18 years old) is overall healthy.

### 3.2. Initial Diagnostic Tests

Several complementary diagnostic tests were performed over the course of the patient’s life; however, none resulted in a definitive diagnosis.

**Figure 1:**
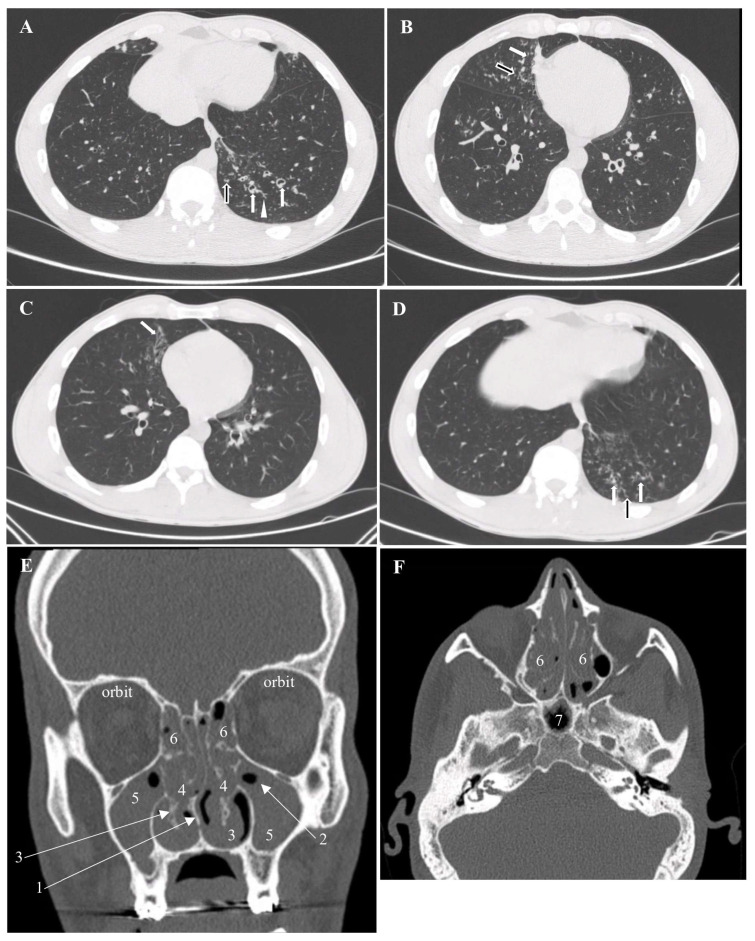
Computed tomography (CT) scan of the chest and paranasal sinuses in the patient. (**A**) Axial view of chest CT scan performed in February 2023, showing the presence of bronchiectasis (white arrows), micronodules (black arrow), peribronchovascular thickening, and tree in bud (white arrowhead) in the lung’s left lower lobe. (**B**) Axial view of chest CT scan performed in February 2023, showing the presence of bronchiectasis (black arrow), micronodules, peribronchovascular thickening, and tree in bud (white arrow) in the lung’s middle lobe. (**C**) Axial view of chest CT scan performed in August 2023, showing the presence of bronchiectasis and peribronchovascular thickening (white arrow) in the lung’s middle lobe. (**D**) Axial view of chest CT scan performed in August 2023, showing the presence of bronchiectasis (white arrows), micronodules, peribronchovascular thickening, and tree in bud (black arrow) in the lung’s left lower lobe. (**E**,**F**) Coronal (**E**) and axial (**F**) views of paranasal sinuses CT scan of the patient performed in February 2023, showing nasal polyposis and inflammatory pansinus disease; blockage of the ostiomeatal complexes; dextroconvex deviation of the nasal septum with a right parasagittal bone spur; inferior turbinate hypertrophy; partial obliteration of the nasal fossae; hypertrophy of the nasopharyngeal lymphatic tissue; sclerodiploic mastoids; and inflammatory filling of the remaining mastoid cells and the right tympanic cavity. (1) Nasal septum, (2) ostiomeatal complex, (3) inferior turbinate, (4) middle turbinate, (5) maxillary sinus, (6) ethmoid sinus, and (7) sphenoid sinus.

A high-resolution chest CT scan revealed bronchiectasis, predominantly affecting the middle and lower lobes, associated with peribronchovascular thickening, findings consistent with an inflammatory process (Figure 1A–D). Computed tomography (CT) of the paranasal sinuses demonstrated nasal polyposis and inflammatory pansinusitis with obliteration of the ostiomeatal complexes (Figure 1E,F).

Pulmonary function testing was normal, with a forced expiratory volume in one second (FEV1) of 87% of the predicted value, and a negative bronchodilator response. Complete blood count was within normal limits, with no peripheral eosinophilia. Biochemical evaluation, including lipid and glucose profiles, as well as renal and hepatic function tests, was unremarkable. Serological testing was normal. Serum alpha-1-antitrypsin levels, antinuclear antibodies, and immunoglobulins (IgA, IgM, and IgE) were within reference ranges. Total serum total IgG was mildly elevated (1870 mg/dL; reference range, 650–1500 mg/dL), as were the IgG subclasses for IgG4 (173 mg/dL; reference range, 7–89 mg/dL) and IgG2 (970 mg/dL; reference range, 124–549 mg/dL) levels. Specific IgE levels for *Aspergillus fumigatus*, *Aspergillus niger*, and *Candida albicans* were normal. Following systemic symptoms after bee stings, the patient was evaluated for suspected Hymenoptera venom allergy, which was confirmed by elevated *Apis mellifera*-specific IgE levels.

Given a history of difficulty in conceiving, semen analysis was performed according to WHO guidelines [46]. The ejaculate showed normal volume, reduced total sperm count, sperm concentration, and sperm normal morphology, but with normal total sperm motility and sperm progressive motility. Overall, these findings were consistent with severe oligoteratozoospermia with preserved sperm motility. The patient was subsequently referred for consultation in a medical-assisted reproduction public hospital unit.

### 3.3. Targeted Diagnostic Workup

Although the proband presented a PICADAR score of 4 [47], which is below the threshold typically associated with a high probability of PCD, he evidenced multiple additional clinical features suggestive of PCD, and thus further targeted diagnostic investigations were pursued. An nNO measurement could not be performed. Cystic fibrosis (CF) was considered unlikely, as the sweat chloride concentration was 19 mmol/L.

#### 3.3.1. High-Speed Video Microscopy

The patient was submitted to nasal brushing for HSVM and TEM analysis. The sample presented a reduced number of cell clusters and of ciliated cells. Quantitative digital HSVM analysis revealed a null CBF mean and a fully dyskinetic CBP, with 100% of the ciliary movements being of the immotile type (Supplementary Videos).

#### 3.3.2. Transmission Electron Microscopy

**Figure 2:**
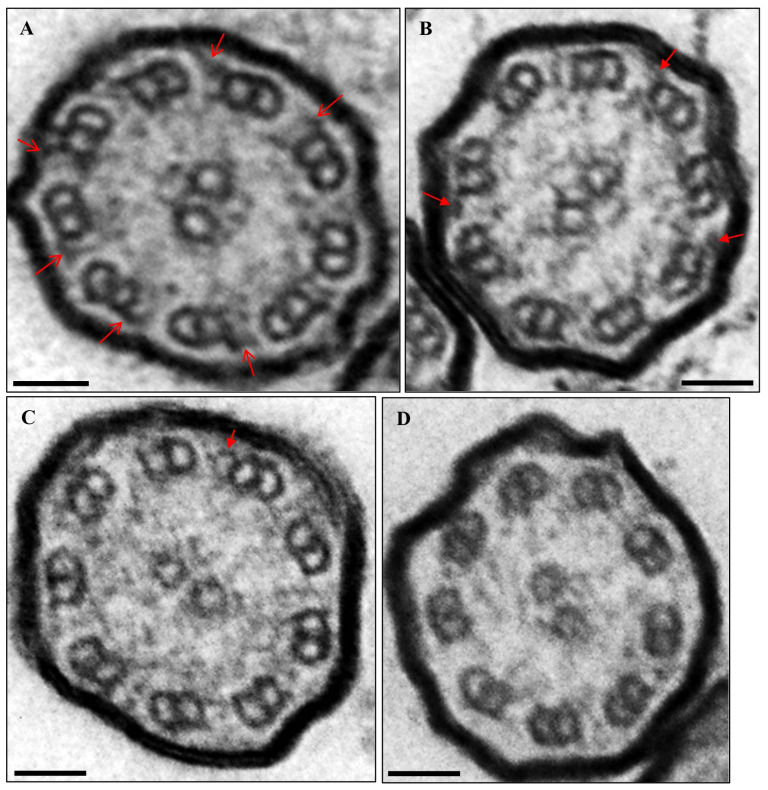
Ultrastructural images of the axoneme from nasal ciliated cells of the proband. Arrows indicate dynein arms that serve as a template for this axoneme. According to international guidelines, a normal axoneme must contain at least 5 clearly visible outer dynein arms (ODAs) and 3 inner dynein arms (IDAs), with inner dynein arms frequently being less defined. Normal ODA are indicated by the red arrows. (**A**) Control (normal) axoneme: Absent, decreased size, or poorly defined ODA *n* = 3 (pathological ≥ 5). (**B**) Patient results: absent, decreased size, or poorly defined ODA *n* = 6 (pathological ≥ 5). (**C**) Patient results: Absent, decreased size, or poorly defined ODA *n* = 8 (pathological ≥ 5). (**D**) Patient results: Absent, decreased size, or poorly defined ODA *n* = 9 (pathological ≥ 5). Scale bar: 50 nm.

Quantitative TEM studies showed 14.8% of inflammatory signs, indicating that inflammatory changes did not compromise TEM interpretation; 56.6% of misaligned basal bodies (Supplementary Figure S1); 25.9% of central pair defects (Supplementary Figure S2); and 100% of the axonemes with absence, reduced dimension, or poorly defined ODA, thus belonging to class-1 defects [30], which is indicative of PCD. The determination of the ciliary beat axis deviation revealed a value of 36.8%, compatible with the distribution described in patients with a ciliary movement disorder such as PCD [42] (Figure 2). We could not confirm these observations after ALI culture, as this technique is still not available.

#### 3.3.3. Genetic Testing by Whole-Exome Sequencing

**Figure 3:**
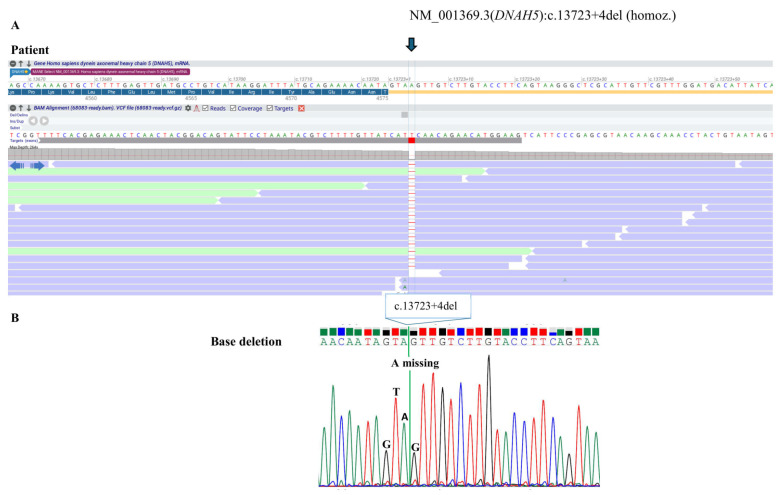
Sequencing results depicting c.13723+4del variant. (**A**) Visualization of the WES data for the *DNAH5* gene region depicting the c.13723+4del homozygous variant. The BAM file is displayed using Alamut Visual software, version 2.0.1, showing read alignment and coverage at the variant site, according to the gene structure. (**B**) Sanger sequencing electropherogram confirming the presence of the c.13723+4del homozygous variant in *DNAH5*. The deletion is indicated by the absence of the peak corresponding to an A base.

**Figure 4:**
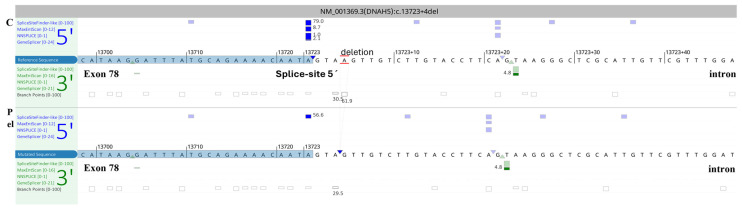
Bioinformatic prediction of the c.13723+4del homozygous variant effect over splicing. In silico analysis was performed to assess the impact of the c.13723+4del variant on pre-mRNA splicing using the algorithms included in Alamut Visual, version 2.0.1, software. The prediction indicates a loss of the canonical donor splice site in intron 78, which is expected to disrupt normal splicing and potentially lead to exon 78 skipping or activation of cryptic splice sites. C-control; P-proband.

Following these results, peripheral blood was collected for genetic analysis by WES. The proband evidenced a new homozygous frame shift variant in the *DNAH5* gene, NM_001369.3(*DNAH5*):c.13723+4del, classified as of unknown clinical significance (Figure 3), with bioinformatic analysis predicting to alter the splicing mechanism (Figure 4).

Only the homozygous variant in *DNAH5* locus was identified during the analysis of the PCD-related gene list. No other variants, including pathogenic/likely pathogenic or hot-VUS were detected.

The identified variant is characterized by deletion of a nucleotide base (adenosine) at position 13723+4. The *DNAH5* gene has 79 exons, and the deletion position occurs at the donor splice site 5′ of intron 78. Bioinformatic tools suggested that the donor splice site of intron 78 becomes non-recognizable by the spliceosome machinery, thus causing the removal of exon 78 (exon 78 skipping) or a usage of an alternative cryptic splice-site. Such alterations would produce a shorter DNAH5 mRNA transcript. At least for the exon 78 skipping, it was predicted to alter the reading frame, introducing a premature codon stop. As a consequence, the mRNA would suffer decay (with decreased or absent protein synthesis), and/or translation would occur with the synthesis of a truncated protein, which would be expected to be non-functional and/or associated with cilium misdirecting.

#### 3.3.4. mRNA Sequencing

**Figure 5:**
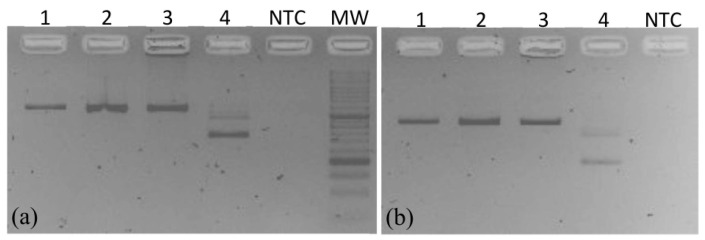
RT-PCR amplification for *DNAH5* at the mRNA level obtained from nasal epithelial cells using two different primer pairs. (**a**) RT-PCR analysis of the DNAH5 transcripts between exons 76 to 79; lanes 1, 2, and 3 correspond to control samples (565 bp), and lane 4 corresponds to the patient’s sample (464 bp and 333 bp). NTC represents the no-template control. MW, molecular weight marker. (**b**) RT-PCR analysis of the DNAH5 transcripts between exons 77 and 79; lanes 1, 2, and 3 correspond to control samples (431 bp), and lane 4 corresponds to the patient’s sample (332 bp and 199 bp). NTC represents the no-template control.

**Figure 6:**
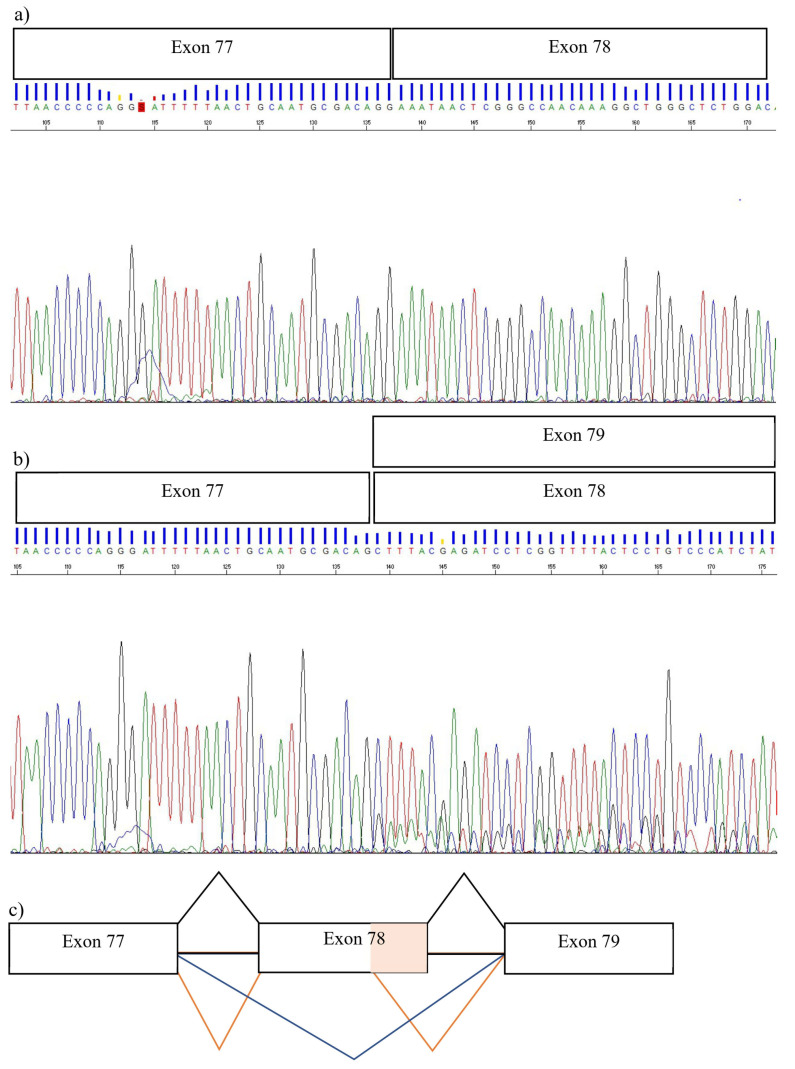
RT-PCR sequencing results for DNAH5 at the mRNA level obtained from nasal epithelial cells using two different primer pairs. (**a**) Electropherogram of the control sample, showing the transition between exons 77 and 78. (**b**) Electropherogram of the patient sample, showing a more represented transcript, in which exon 79 follows exon 77 (skipping of exon 78), and a minor transcript, in which exon 78 follows exon 77 (in this transcript, the last 99 nucleotides of exon 78 are deleted, but this is not visible in this electropherogram). (**c**) Schematic representation of the splicing pattern observed between exons 77 and 79; at the top, in black, the normal splicing process is represented; at the bottom, in blue, splicing with skipping of exon 78 (most prevalent transcript) is represented, and in orange, the splicing pattern in which the last 99 nucleotides of exon 78 (less represented transcript) are eliminated.

As WES findings indicated a splice defect, proband ciliated cells were processed for mRNA extraction and sequencing. Sequencing of mRNA revealed that the variant led to an alteration of the normal splicing process. Two aberrant transcripts were found: one of them was more prevalent, with skipping of exon 78 [r.13492_13723del p.(Glu4498Leufs*21)], and the other was less present, with deletion of the last 99 base-pairs of exon 78 [r.13625_13723del p.(Gly4542_Thr4575delinsAla)], indicating the introduction of a cryptic splice site in exon 78 (Figure 5 and Figure 6).

#### 3.3.5. Immunofluorescence Analysis of Ciliated Cells and Spermatozoa

**Figure 7:**
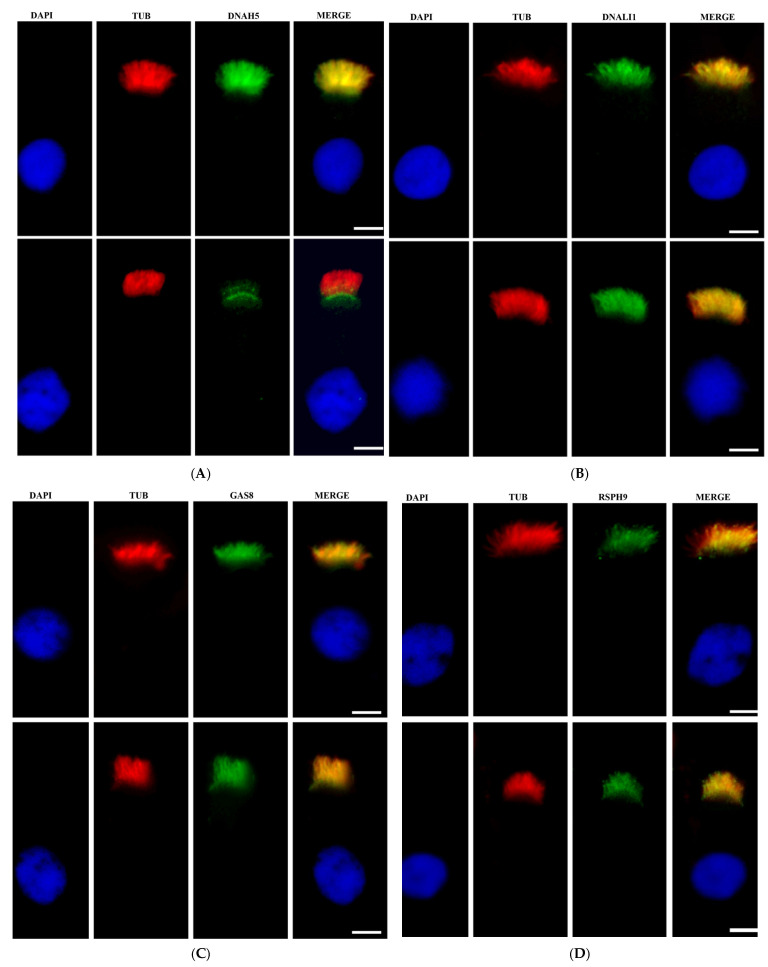
Immunofluorescence analysis of respiratory epithelial cells from the proband. Each panel shows staining for a different axonemal protein: (**A**) DNAH5, component of the outer dynein arms (ODA); (**B**) DNALI1, component of the inner dynein arms (IDA); (**C**) GAS8, component of the nexin–dynein regulatory complex (NDRC); and (**D**) RSPH9, component of the radial spokes (RSs). Cells were stained with DAPI (blue, nuclei), anti-acetylated tubulin (TUB, red, ciliary axonemes), and antibodies against the corresponding axonemal protein (green). In all panels, the Merge (yellow) images showed co-localization. Fluorescence localization of DNALI1 (for IDA), GAS8 (for NDRC), and RSPH9 (for RS) was preserved and comparable to expected control patterns. In contrast, the DNAH5 signal was absent from the proximal cilia region, appearing concentrated at the transition zone, basal-body region, and base of basal-bodies.

**Figure 8:**
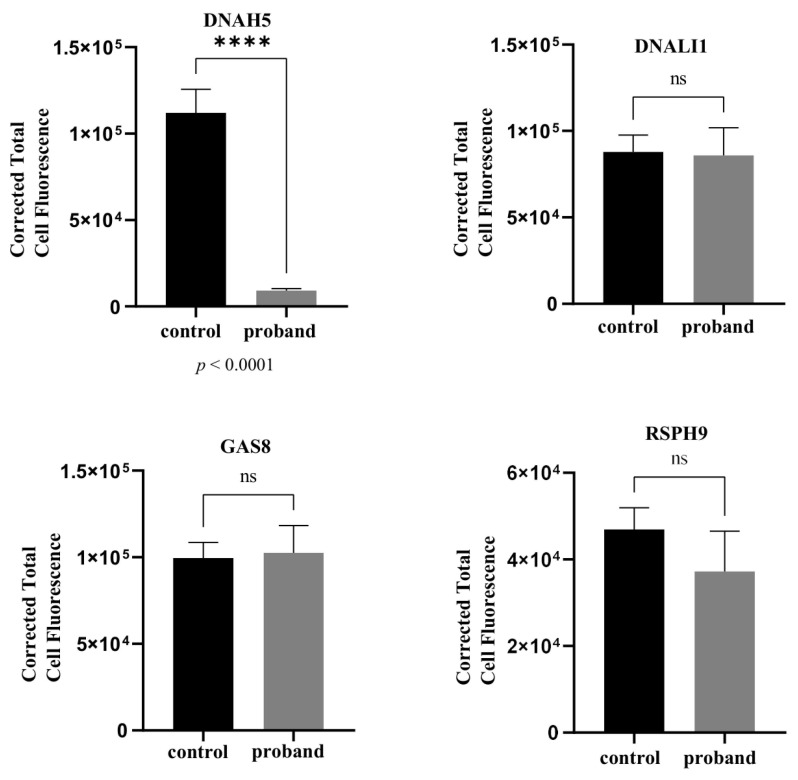
Statistical analysis of the mean fluorescence intensity of respiratory epithelial cells from the proband, using the statistical Mann–Whitney test, taken from the immunofluorescence analysis of proband ejaculated spermatozoa. Corrected total cell fluorescence was calculated from the measurements obtained from the ImageJ software. Statistical significance was set at an alpha value of <0.05. Fluorescence intensity of DNALI1 (for inner dynein arms), GAS8 (for nexin–dynein regulatory complex) and RSPH9 (for radial spokes) antibodies was preserved and comparable to expected control patterns. In contrast, DNAH5 (for outer dynein arms) fluorescence intensity was markedly reduced. This reduction was statistically significant, based on the comparison of mean fluorescence intensity in control versus the proband samples, and is consistent with selective loss of outer dynein arms components. *p*-values: DNAH5 (*p* < 0.0001); DNALI1 (*p* = 0.6970); GAS8 (*p* = 0.3155); RSPH9 (*p* = 0.0598). Statistical significance is indicated as **** for *p* < 0.0001.

To further investigate the impact of the identified variant, IF was performed on nasal ciliated cells, and it revealed the absence of the DNAH5 protein along the axoneme, with concentration at the cilium neck and basal body regions. No changes in immunofluorescence intensity and spatial localization were identified in the tested proteins, related to the inner dynein arms (IDA), nexin–dynein regulatory complexes (NDRCs), or radial spokes (Figure 7 and Figure 8).

**Figure 9:**
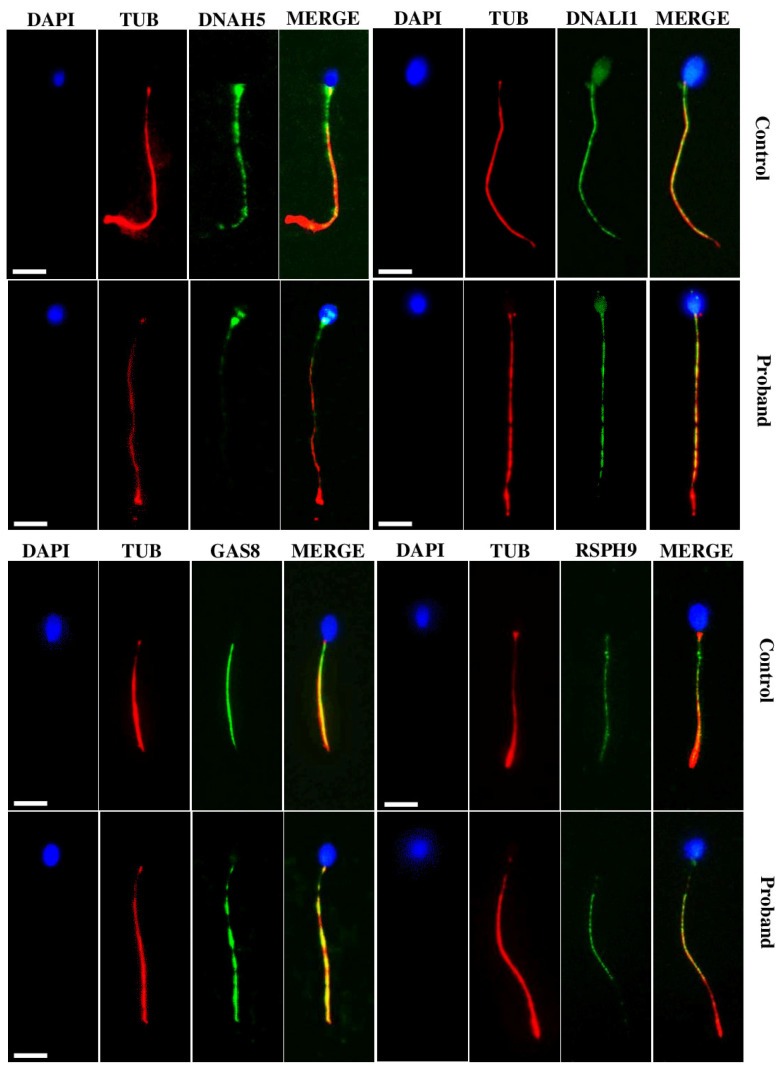
Immunofluorescence analysis of spermatozoa from the proband. Each panel shows staining for a different axonemal protein: DNAH5, component of the outer dynein arms (ODA); DNALI1, component of the inner dynein arms (IDA); GAS8, component of the nexin–dynein regulatory complex (NDRC); and RSPH9, component of the radial spokes (RSs). Cells were stained with DAPI (blue, nuclei), anti-acetylated tubulin (TUB, red, ciliary axonemes), and antibodies against the corresponding axonemal protein (green). In all panels, the Merge (yellow) images showed co-localization. Fluorescence localization of DNALI1 (for IDA), GAS8 (for NDRC), and RSPH9 (for RS) was preserved and comparable to expected control patterns. In contrast, the DNAH5 signal was absent in the principal-piece and end-piece, reduced in the midpiece, and concentrated at the neck region.

**Figure 10:**
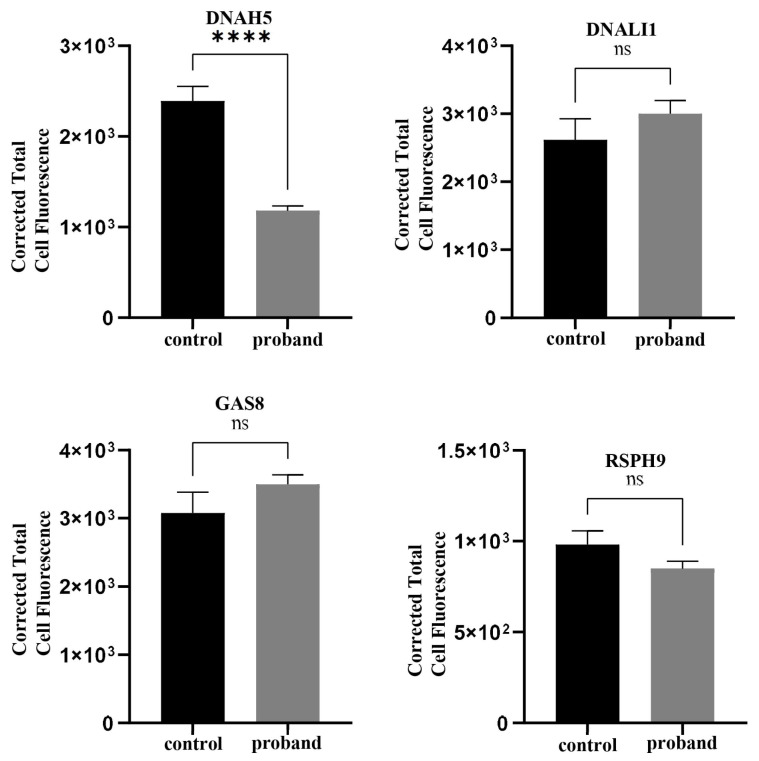
Statistical analysis of the mean fluorescence intensity of axoneme proteins, using the statistical Mann–Whitney test, taken from the immunofluorescence analysis of the proband respiratory epithelial cells. Corrected total cell fluorescence was calculated from the measurements obtained from the ImageJ software. Statistical significance was set at an alpha value of <0.05. Fluorescence intensity of DNALI1 (for inner dynein arms), GAS8 (for nexin–dynein regulatory complex) and RSPH9 (for radial spokes) antibodies was preserved and comparable to expected control patterns. In contrast, DNAH5 (for outer dynein arms) fluorescence intensity was markedly reduced. This reduction was statistically significant (*p* < 0.0001), based on the comparison of mean fluorescence intensity in control versus the proband samples, and is consistent with selective loss of outer dynein arms components. *p*-values: DNAH5 (*p* < 0.0001); DNALI1 (*p* = 0.3293); GAS8 (*p* = 0.0964); RSPH9 (*p* = 0.1161). Statistical significance is indicated as **** for *p* < 0.0001.

Analysis of spermatozoa by IF revealed absence of DNAH5 staining in the axoneme, with accumulation at the sperm neck region (Figure 9 and Figure 10).

### 3.4. Family Studies

**Figure 11:**
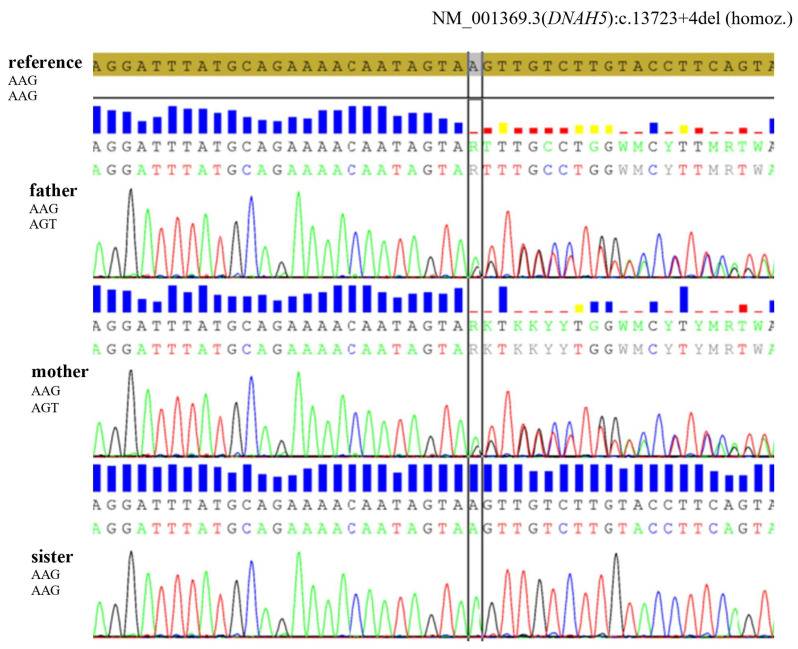
Sanger sequencing of the *DNAH5* c.13723+4del variant in the patient’s family members. Electropherograms obtained by Sanger sequencing confirm the presence of the c.13723+4del variant in the patient’s relatives. The analysis shows that both parents are heterozygotes, consistent with an autosomal recessive inheritance pattern. The sister presents two normal *DNAH5* alleles.

**Figure 12:**
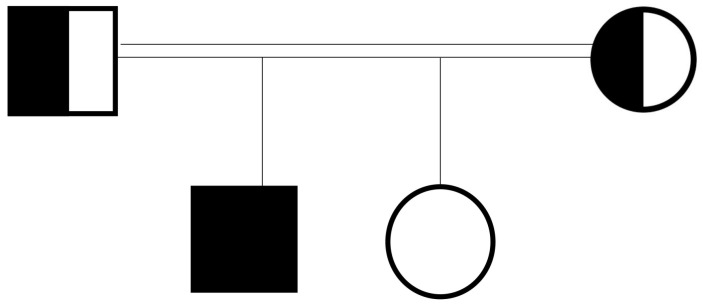
Family pedigree illustrating segregation of the *DNAH5* c.13723+4del variant. The pedigree depicts the proband, parents (consanguineous), and sibling, indicating the genotype of each individual. The segregation analysis demonstrates that the variant follows an autosomal recessive inheritance. The black color indicates the allele containing the variant, with parents being heterozygous and the son homozygous for the variant.

**Figure 13:**
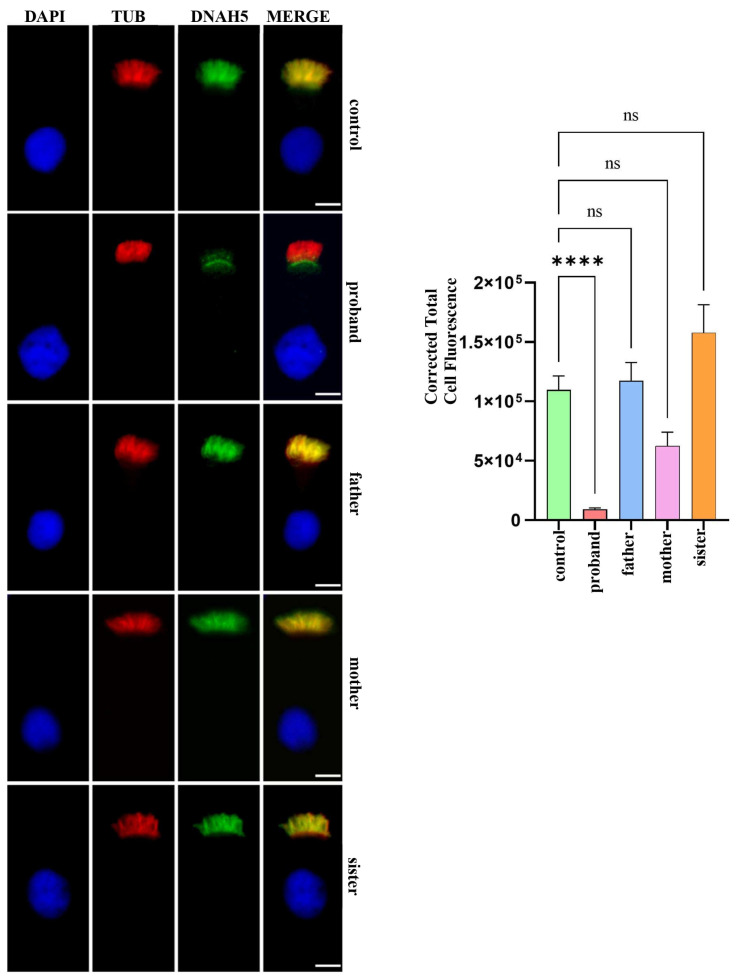
Immunofluorescence analysis of respiratory epithelial cells from the proband and family members. Cells were stained with DAPI (blue, nuclei), anti-acetylated tubulin (TUB, red, ciliary axonemes), and the axonemal protein DNAH5 (green). In all panels, the Merge (yellow) images showed co-localization. Fluorescence localization of DNAH5 was preserved and comparable to expected control patterns in proband parents and sister. Statistical analysis of the mean fluorescence intensity of axoneme proteins, using the statistical test Kruskal–Wallis for multiple comparisons, taken from the immunofluorescence analysis of the proband and family members respiratory epithelial cells. Corrected total cell fluorescence was calculated from the measurements obtained from the ImageJ software. Statistical significance was set at an alpha value of <0.05. Fluorescence intensity of DNAH5 (for outer dynein arms) was preserved and comparable to expected control patterns in proband parents and sister. *p*-values: controls vs. patient (*p* < 0.0001); controls vs. father (*p* = 0.6848), controls vs. mother (*p* = 0.1503); controls vs. sister (*p* = 0.1324). Statistical significance is indicated as **** for *p* < 0.0001. ns: not statistically significant.

Sanger sequencing of first-degree family members revealed that the variant was inherited from the father and mother, whereas the sister presented normal alleles (Figure 11). The parents presented first-degree consanguinity (Figure 12). The analysis by HSVM revealed decreased CBF and normal CBP, and IF analysis showed a normal presence of the DNAH5 protein (Figure 13).

## 4. Discussion

In this report, we described a patient from a consanguineous family presenting with chronic rhinosinusitis, chronic otitis media, and bronchiectasis, with recurrent infections since infancy. After excluding other respiratory diseases [48,49,50,51], a clinical suspicion of PCD was raised. This was supported by HSVM [29,37] and TEM [30,42] findings, showing absent ciliary beating and loss of outer dynein arms. Genetic analysis identified a novel homozygous frameshift variant in *DNAH5*, with immunofluorescence [33,34,36] confirming absent protein in the apical cilium. Semen analysis [46] revealed severe oligoteratozoospermia with preserved motility, consistent with the non-involvement of DNAH5 in sperm motility [52,53,54]. The relatively low PICADAR score [47] and absence of laterality defects contributed to a delayed diagnosis at 23 years, while family studies confirmed parental heterozygosity and normal ciliary function.

In ciliated cells, the ODA exhibit two subtypes, ODA1 (DNAH5+DNAH11) in the proximal (basal) axoneme region (cilium neck, which contains the transition zone and the distal (apical) microtubule organizer center that polymerizes the central microtubule pair), and ODA2 (DNAH5+DNAH9) in the distal axoneme region [55,56,57,58,59].

In a previous study, DNAH5 was observed at the sperm axoneme proximal region, and DNAH9 along the whole axoneme [13]. In another study, the function of the sperm DNAH5 was suggested to be replaced by ODAH8 and DNAH17, which were present all over the entire sperm axoneme, contrary to DNAH5, DNAH9, and DNAH11, which were absent [52]. Other authors also observed that DNAH5 was totally absent from the sperm axoneme, with DNAH8 being observed all along the axoneme [54]. The present report showed the presence of DNAH5 all along the axoneme of control spermatozoa, whereas in the mutated case, staining became restricted to the sperm neck region. At the moment, we cannot explain these staining discrepancies regarding DNAH5 in spermatozoa. Fliegauf et al. [55] used a monoclonal antibody, and the “proximal” region of the sperm axoneme was stained; Aprea et al. [54] used the same antibody and observed no staining; and Whitfield et al. [52] and our team used the same polyclonal antibody, and, whereas the former obtained no staining, our results showed axoneme labeling. Nevertheless, the important conclusion is that, present or not present, we here confirm that *DNAH5* biallelic mutations have no effect on sperm motility.

The Dynein Axonemal Heavy Chain-5 gene (*DNAH5*) (OMIM number: 608644) is one of the most frequently implicated genes in PCD, with genetic defects causing PCD type-3, Kartagener syndrome, and absence of the outer dynein arms [60]. The ODA encodes a dynein protein, which is a microtubule-associated motor protein complex consisting of heavy (α: DNAH5; β: DNAH9 distal and DNAH11 proximal, and, in sperm, DNAH8 and DNAH17), light, and intermediate chains. In ciliated cells, DNAH5 functions as a force-generating protein with ATPase activity, whereby the release of ADP is thought to produce the force-producing power stroke [61,62]. Genetic defects in *DNAH5* typically lead to absent or dysfunctional ODA, resulting in impaired mucociliary clearance and the characteristic clinical features of PCD [60,63], with biallelic pathogenic variants in *DNAH5* accounting for a substantial proportion of genetically confirmed PCD cases [55,64].

The *DNAH5* gene contains 79 exons and is localized at chromosome 5p15.2 [60,63], and protein DNAH5 contains 4624 amino acids. The ODA is made of two dynein heavy chains, two intermediate chains, and eight light chains [59]. The dynein heavy chain contains domains for ATP binding and hydrolysis, and for microtubule binding. The dynein intermediate and light chains interact with heavy chain domains, assisting in motor regulation [65]. Structurally, DNAH5 presents an N-Terminal tail region, with domains for the assembly and docking of the intermediate and light chains, followed by a linker domain that arches over a globular head, enabling force movement generation. The globular head (motor domain) is a hexameric ring that is responsible for ATP hydrolysis, enabling transformation of chemical energy into mechanical motion and force. Motor domains 3–4 bind ATP, and motor domain 1 hydrolyzes ATP. Domain 2 behaves as a regulatory site offering structural stability. Motor domain 4 is then followed by a coiled-coil segment creating a stalk projection. The stalk forms a necklace, possessing a globular microtubule binding domain at the middle region, and then, turning to the globular region, continues with motor domain 5. Motor domain 5 also interacts through a second coiled-coil segment (the buttress) with the stalk. Motor domain 5 is followed by motor domain 6, which ends in the C-terminus. The C-terminus interacts with the linker domain to create conformational changes, producing the force for the power stroke, besides working as a structural stabilization element [60,65,66,67,68,69,70,71,72].

Protein DNAH5 is synthesized in the cytoplasm [68,73], transported to the basal body [74], and then brought to the transition zone [75,76]; there, it joins the other heavy, intermediate, and light chains to form ODA precursors (preassembly); the ODA then associates (loading) with intra-flagellar transport particles, being transported and integrated (final assembly) in the peripheral doublet A-microtubule of the axoneme [77,78].

Disease-causing variants in the *DNAH5* gene disrupt the assembly and transport of the ODA due to protein misfolding or incorrect loading, which lead to accumulation at the ciliary base and basal-body, with absent integration or malfunction in the outer dynein arms [60,63,64].

In the present case, immunofluorescence revealed the absence of DNAH5 at the distal region, remaining, although with decreased intensity, accumulated at the transition zone, in the basal-body region, and at the base of the basal-body, suggesting a similar mechanism to that formerly described [60,63,64]. In the ultrastructural examination, the patient showed, in the majority of the axonemes, absence of five or more, with this number being sufficient to belong to class-1 [30] and cause cilia immotility. However, this also means that some ODAs are still present, which could explain the residual protein levels in the preassembly region of the transition zone.

**Figure 14:**
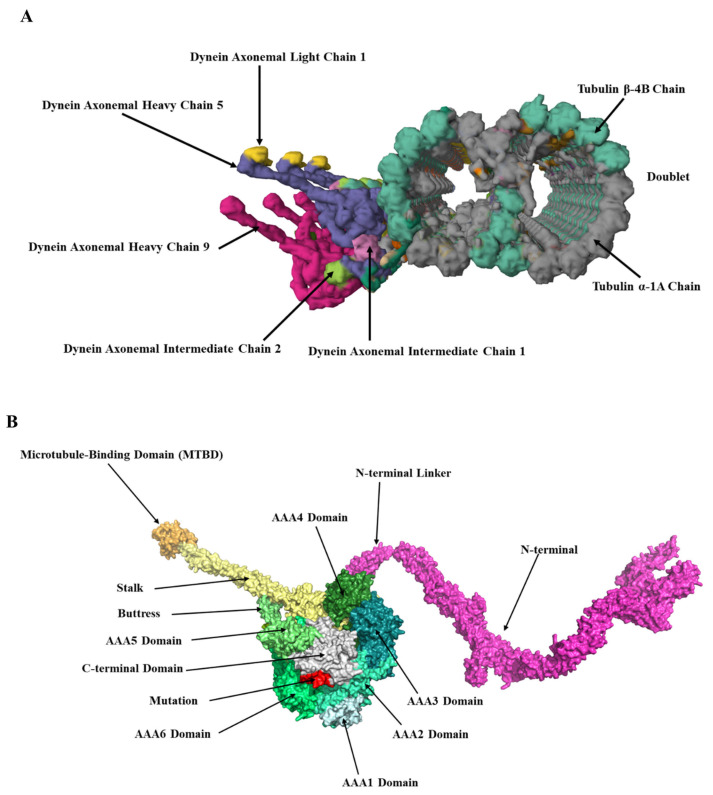
Structural Protein Analyses. Structural models obtained by X-ray crystallography and cryo-electron microscopy from multiple sources and available in the Protein Data Bank were analysed. These included models of human and DNAH5 dynein axonemal heavy chains, as well as non-human axonal structures. (**A**) Cross-section of the human respiratory doublet microtubule and associated outer dynein arm (structural model PDB ID: 8J07 [79]). (**B**) Model of DNAH5, coloured by the structural elements identified in the figure, with the C-terminal mutation position highlighted in red. (**A**) was prepared using Mol* [80] and (**B**) using PyMOL, version 3.1 [81].

The here-studied variant is characterized by deletion of a nucleotide base (adenosine) at position 13723+4. This deletion position occurs at the donor splice site 5′ of intron 78. This region corresponds to the C-terminal subdomain of DNAH5. Sequencing of mRNA revealed that the variant c.13723+4del leads to abnormal splicing, with two aberrant transcripts being found, one more prevalent, with skipping of exon-78 [r.13492_13723del p.(Glu4498Leufs*21)], and the other, less present, with deletion of the last 99 base-pairs of exon 78 [r.13625_13723del p.(Gly4542_Thr4575delinsAla)], suggesting the introduction of a cryptic splice site in exon-78. Exon-78 skipping altered the reading frame and introduced a premature stop codon in exon 79, leading to a smaller protein of 4498+21 amino acids. In the cryptic splicing site situation, the protein lost 33 amino acids, with an alanine being inserted in exon-78. This did not cause an altered reading frame or the introduction of a premature stop codon. To our knowledge, these alterations have not been previously reported, with the consequent protein loss of function establishing the pathogenicity of the variant (Figure 14; Supplementary Figure S3).

The structure of the human respiratory doublet microtubule and its associated axonemal complexes was determined by cryogenic electron microscopy, revealing in atomic detail how dynein heavy chains are structurally organized (Supplementary Figure S3). DNAH5 is a massive motor protein that forms the core of the outer dynein arm (ODA), being associated with light and intermediate chains (Figure 14A). Its C-terminal domain is primarily dedicated to intramolecular architecture, performing two vital tasks: first, it loops over the AAA+ hexameric ring to mechanically brace and stabilize the motor domains, preventing them from warping or collapsing under mechanical stress; second, it physically interacts with the AAA1 and AAA2 domains to regulate the conformational changes necessary for the linker arm to pull against microtubules. Without the final amino acids encoded by exons 78 and 79, the DNAH5 motor ring becomes structurally unstable and mechanically inactive (Figure 14B). Consequently, the outer dynein arms cannot complete their assembly or attach to the ciliary axoneme, which clinically manifests as primary ciliary dyskinesia.

Other cases previously reported mutations in the DNAH5 C-terminal region (exons 73–79), also demonstrating that, in these cases, DNAH5 is lost only in the distal region of the cilium axoneme. Hornef et al. [64]. reported several variants in the C-terminal region of DNAH5; in one case (OP40), the insertion of a nucleotide in exon 77 was predicted to cause a frameshift mutation, leading to the introduction of a premature codon stop and a resultant truncated protein; in the other case (F718), which had two intronic variants, the substitution of a nucleotide at the acceptor site of exon 75 and at the donor site of exon 76 was predicted to lead to exon skipping or alternative splicing with the introduction of a premature codon stop and a resultant truncated protein; in both cases, IF showed loss of the distal labeling, with staining remaining at the proximal ciliary region, with authors suggesting that the assembly of the proximal ODA was at least preserved [64]. Ta Shma et al. [82] presented a case with an exonic deletion of four nucleotides in exon 77, with a predicted frameshift occurrence followed by the introduction of a premature codon stop and a resultant truncated protein; also, in this case, the DNAH5 staining was lost only at the distal ciliary region [82]. Van Capelle et al. [62] presented a case with deletion of four nucleotides in exon 76, with a predicted frameshift followed by the introduction of a premature codon stop and a resultant truncated protein; also, in this case, the DNAH5 staining was lost only at the distal ciliary region [62]. Finally, Dong et al. [83] also confirmed that variants attaining the C-terminus cause loss of DNAH5 only at the distal region of the cilium [83].

## 5. Conclusions

This report expands the mutational spectrum of *DNAH5* causing PCD, adding a new homozygous single-nucleotide deletion affecting a donor splice-site. To our knowledge, this is the first case of a PCD patient with such variant type with full characterization at the mRNA and protein level. The collected data also enabled its reclassification from VUS to pathogenic, being of educational value by highlighting the strategies for variant clinical actionability. Our results are expected to add knowledge on PCD disease mechanisms and also underscore the importance of integrating clinical, ultrastructural, molecular, and protein expression analyses to clarify and contribute to PCD diagnosis and a better understanding of the relationships between phenotypes and genotypes in PCD, besides now serving as potential markers for inclusion in genetic diagnostics and future targeted therapies. Furthermore, for the patient and family, it is important to know the exact cause of the disease, and knowing the cause enables clinicians to provide targeted therapy and direct the patient to genetic and reproductive counseling.

## Data Availability

The data that support the findings of this study are available from the corresponding author upon reasonable request.

## Supplementary Materials

The following supporting information can be downloaded at https://www.mdpi.com/article/10.3390/cells15111022/s1, Figure S1: Displacement or misalignment of basal bodies; Figure S2: Ultrastructure of the axoneme showing abnormalities of the central pair; Figure S3: Cross-sections of the human respiratory doublet microtubule and associated outer dynein arms; Videos S1–S4: Videos of ciliated cells from controls taken by highspeed videomicroscopy; Videos S5–S10: Videos of ciliated cells from the proband taken by highspeed videomicroscopy.
