# Supplementary material for: Functional Characterization of a Novel Homozygous DNAH5 Single-Nucleotide Intronic Deletion in a Consanguineous Portuguese Family with Primary Ciliary Dyskinesia
Source: Cells. 2026 Jun 2;15(11):1022. doi: 10.3390/cells15111022 (PMC13256310; doi:10.3390/cells15111022)
Supplement: Supplementary file 1 [file cells-15-01022-s001.zip › Figure S1-BB-clean.pdf]

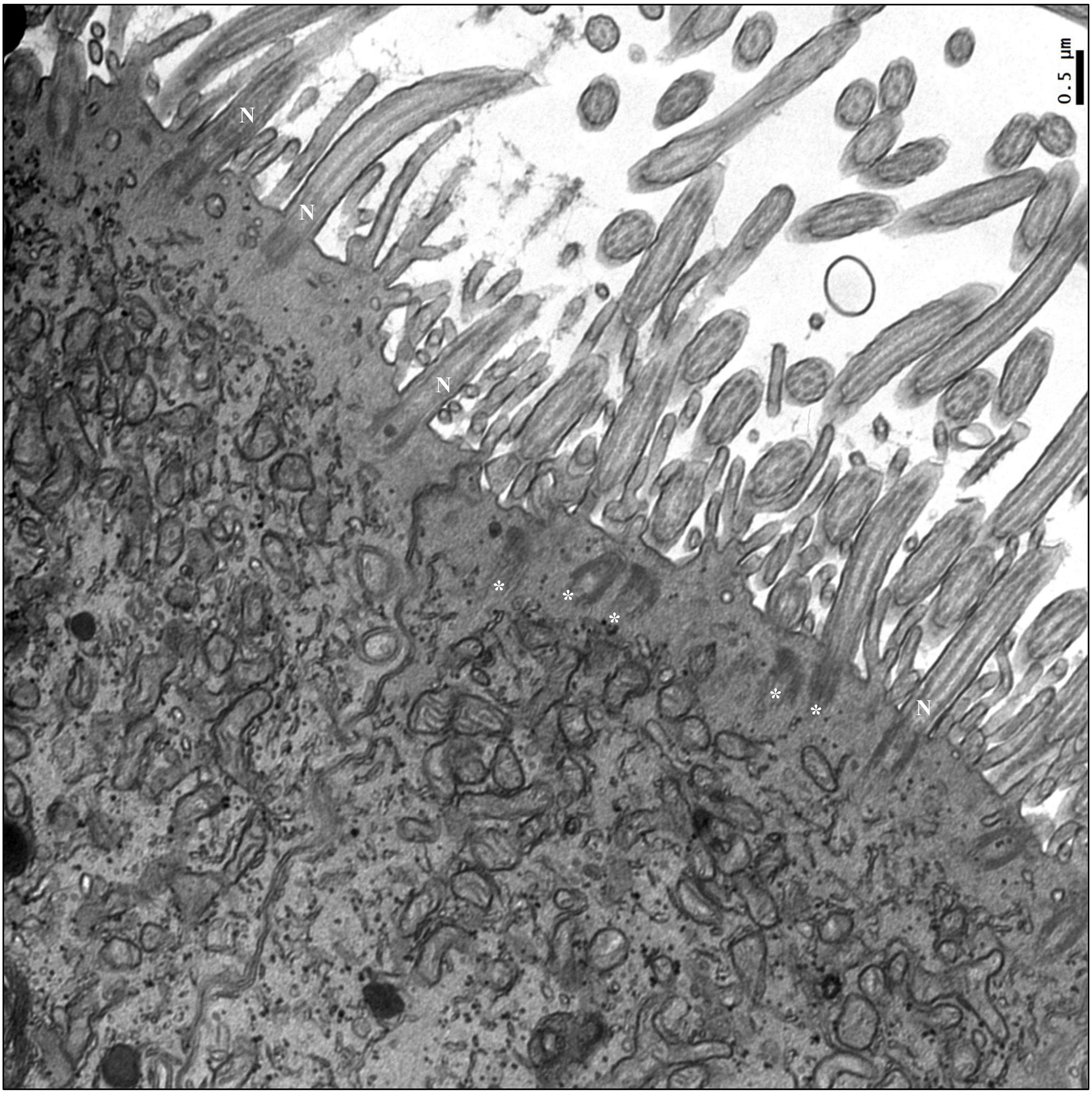

Supplementary Figure S1. **A**. Displacement or misalignment of basal bodies (*) in relation to longitudinally well-orientated cilia axonemes (N).

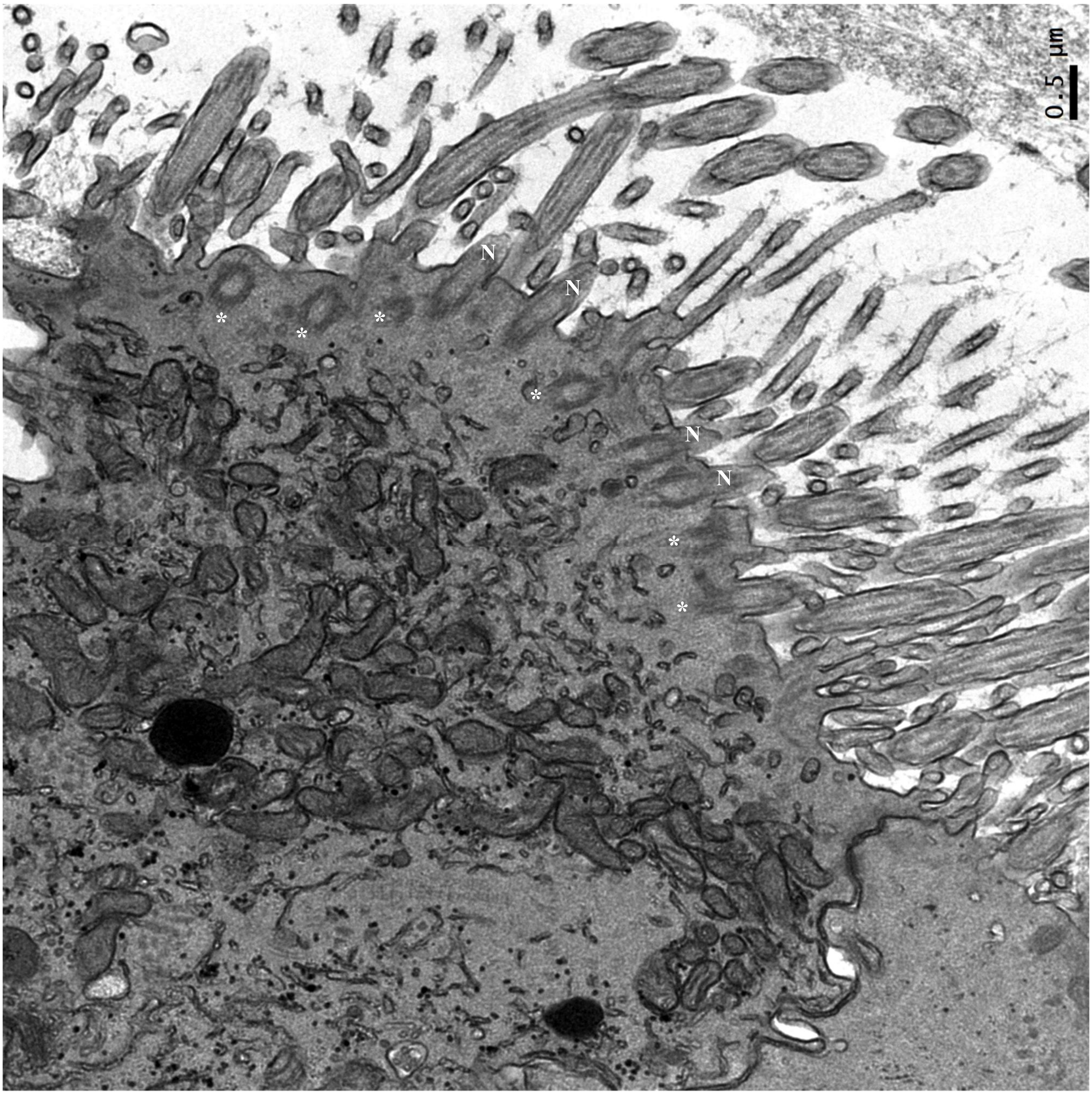

Supplementary Figure S1. **B**. Displacement or misalignment of basal bodies (*) in relation to longitudinally well-orientated cilia axonemes (N).

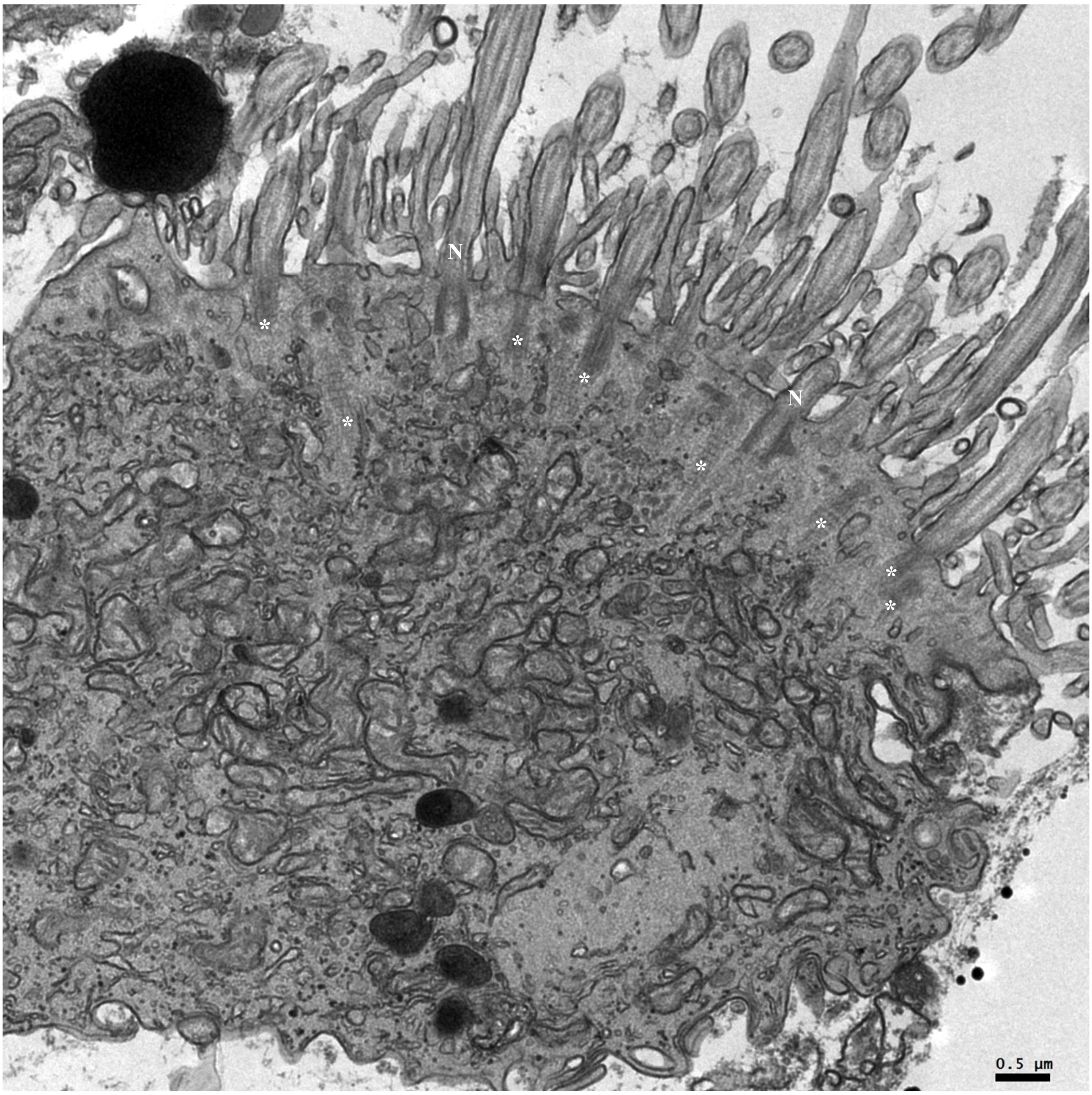

Supplementary Figure S1. **C**. Displacement or misalignment of basal bodies (*) in relation to longitudinally well-orientated cilia axonemes (N).
