# Supplementary material for: Functional Characterization of a Novel Homozygous DNAH5 Single-Nucleotide Intronic Deletion in a Consanguineous Portuguese Family with Primary Ciliary Dyskinesia
Source: Cells. 2026 Jun 2;15(11):1022. doi: 10.3390/cells15111022 (PMC13256310; doi:10.3390/cells15111022)
Supplement: Supplementary file 1 [file cells-15-01022-s001.zip › Figure S2-CP-clean.pdf]

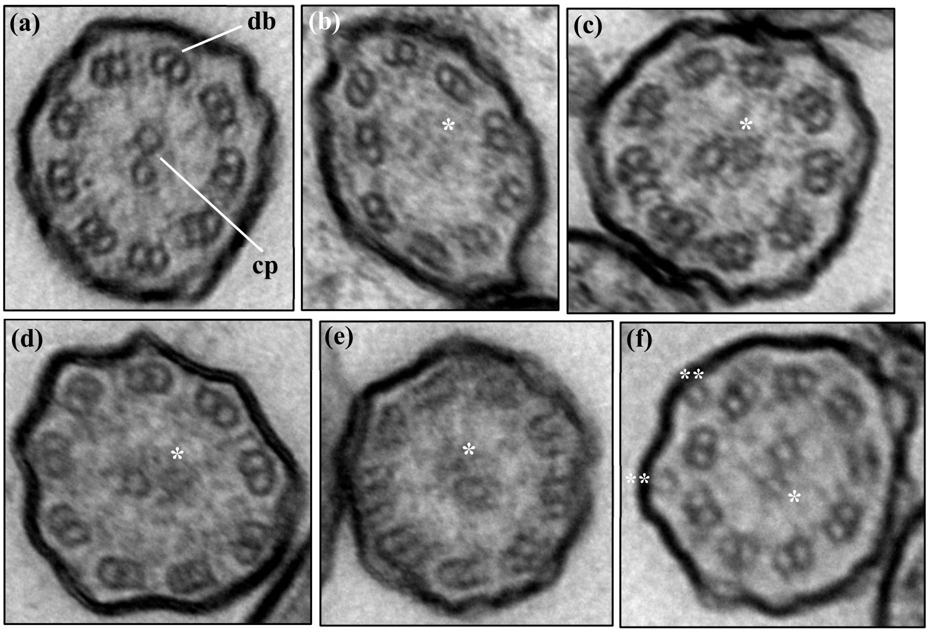

Supplementary Figure S2A. Ultrastructure of the axoneme showing abnormalities of the central pair. **(a)** normal positioning of the peripheral doublets (db) and of the central pair (cp) (equal distance to the peripheral doublets). **(b)** missing central pair. **(c,d)** undefined microtubule of the central pair. **(e)** missing microtubule of the central pair. **(f)** reduced size of one microtubule of the central pair.

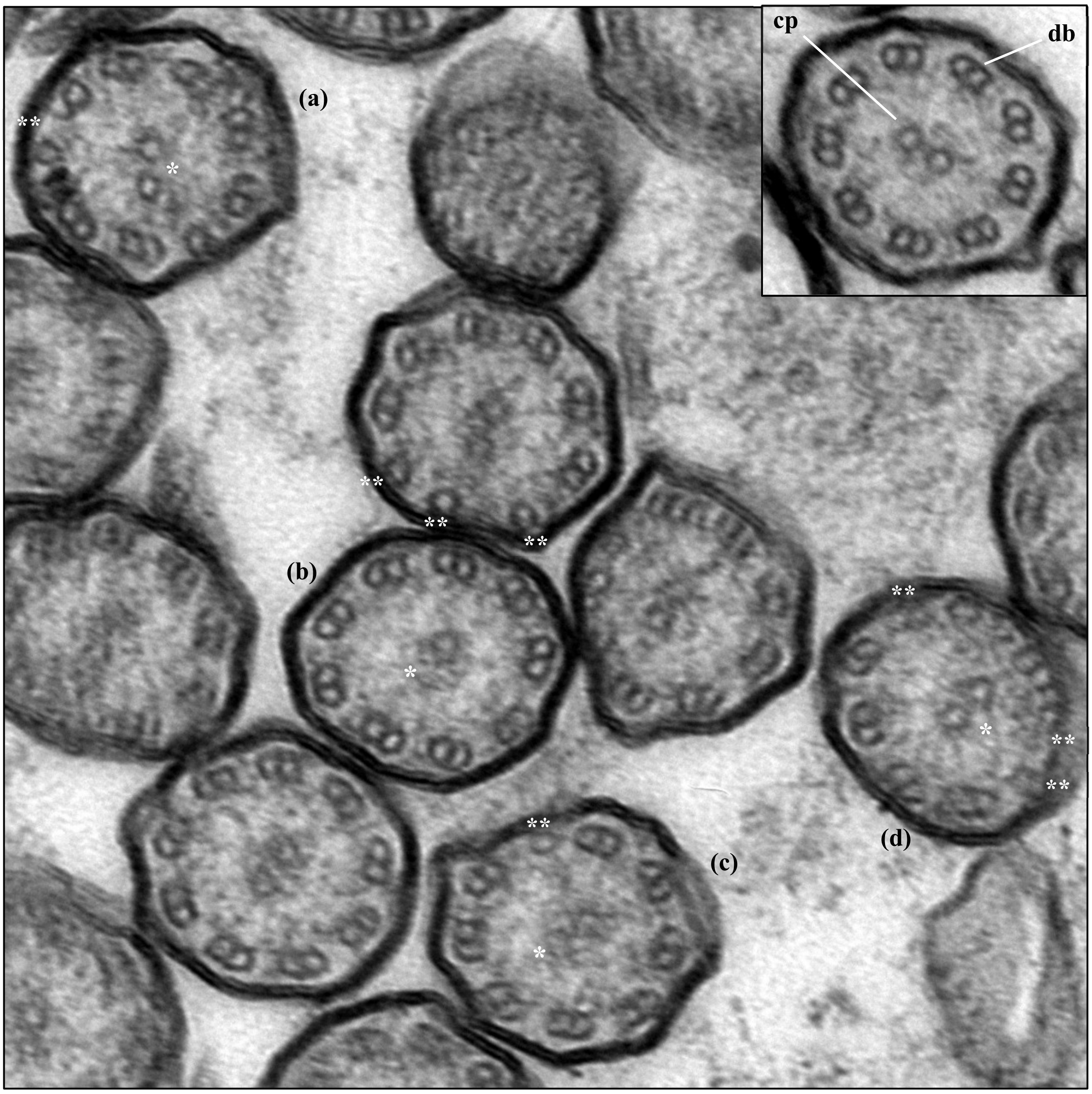

Supplementary Figure S2B. Ultrastructure of the axoneme showing abnormalities of the central pair. **Inset**. Normal positioning of the peripheral doublets (db) and of the central pair (cp) (equal distance to the peripheral doublets). **(a)** displaced central pair. **(b)** missing microtubule of the central pair. **(c)** undefined central pair. **(d)** displaced central pair. (*) abnormalities of the central pair. (**) abnormalities of the peripheral doublets.
