# Supplementary material for: Functional Characterization of a Novel Homozygous DNAH5 Single-Nucleotide Intronic Deletion in a Consanguineous Portuguese Family with Primary Ciliary Dyskinesia
Source: Cells. 2026 Jun 2;15(11):1022. doi: 10.3390/cells15111022 (PMC13256310; doi:10.3390/cells15111022)

Supplementary Figure S3A,B. Cross-sections of the human respiratory doublet microtubule and associated outer dynein arms (structural model PDB ID: 8J07 [79]), prepared using Mol* [80].

A

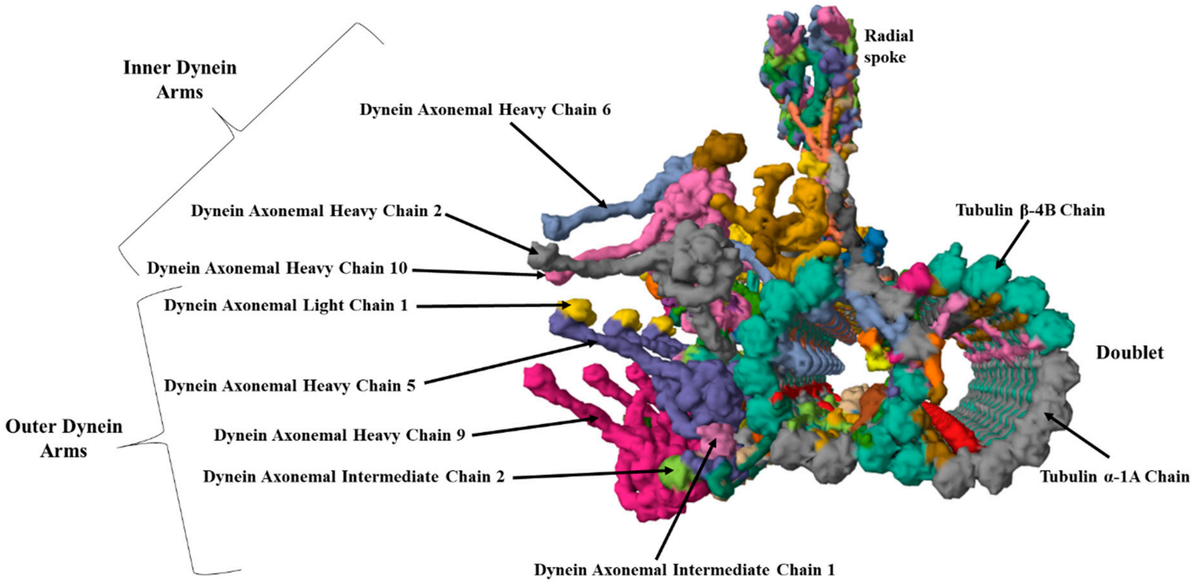

Supplementary Figure S3A. Cross-section of the human respiratory doublet microtubule and associated axonemal complexes, indicating outer and inner dynein arms components.

B

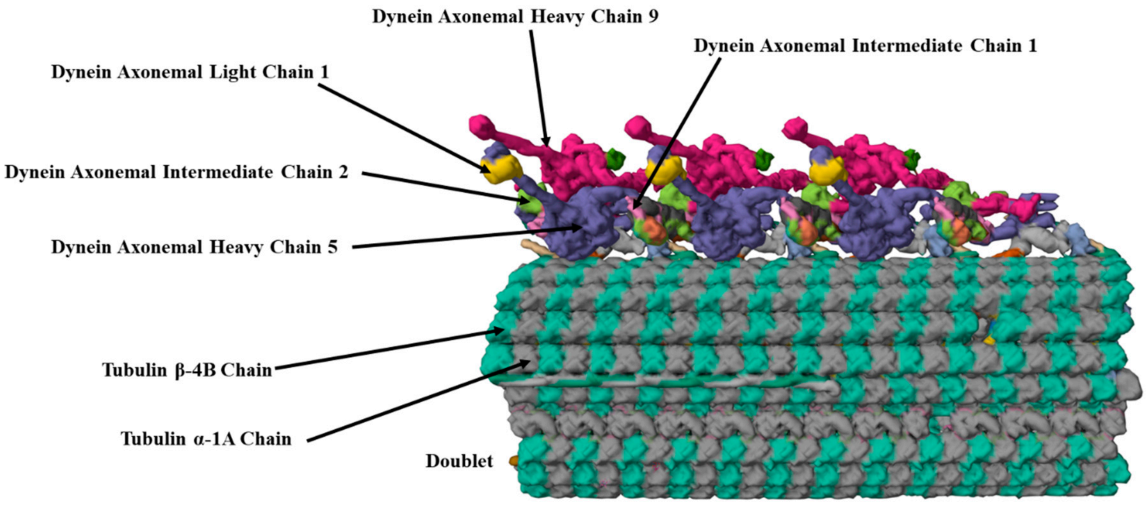

Supplementary Figure S3B. Longitudinal section of the 96-nm repeat of respiratory doublet microtubule and associated outer dynein arms components.

Supplementary Figure S3C. Diagram of DNAH5 domains, evidencing the exons (blue) and aminoacids (red) intervals, depicting the mutation site. Bellow, a DNAH5 model, coloured by the structural elements identified in the figure, prepared using PyMOL [81].

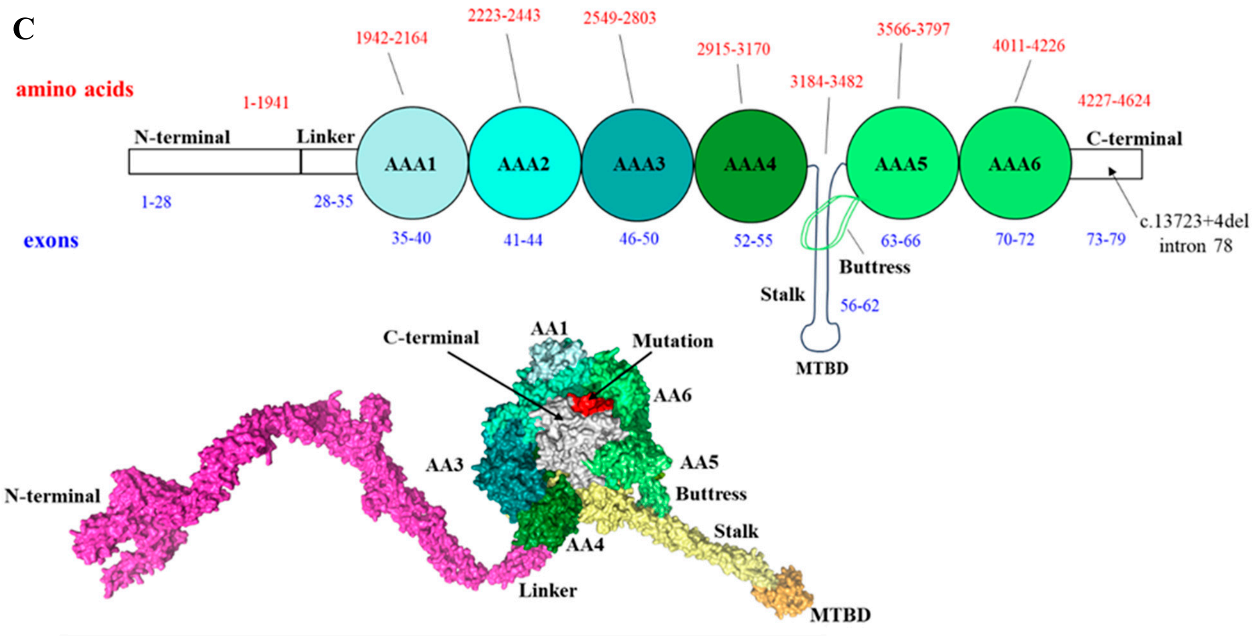

Supplement: Supplementary file 1 [file cells-15-01022-s001.zip › Figure S3-DNAH5-clean.pdf]
